# COVID-19 deaths: Which explanatory variables matter the most?

**DOI:** 10.1371/journal.pone.0266330

**Published:** 2022-04-21

**Authors:** Pete Riley, Allison Riley, James Turtle, Michal Ben-Nun

**Affiliations:** Predictive Science Inc., San Diego, California, United States of America; Shahjalal University of Science and Technology, BANGLADESH

## Abstract

More than a year since the appearance of Severe Acute Respiratory Syndrome Coronavirus 2 (SARS-CoV-2), many questions about the disease COVID-19 have been answered; however, many more remain poorly understood. Although the situation continues to evolve, it is crucial to understand what factors may be driving transmission through different populations, both for potential future waves, as well as the implications for future pandemics. In this report, we compiled a database of more than 28 potentially explanatory variables for each of the 50 U.S. states through early May 2020. Using a combination of traditional statistical and modern machine learning approaches, we identified those variables that were the most statistically significant, and, those that were the most important. These variables were chosen to be fiduciaries of a range of possible drivers for COVID-19 deaths in the USA. We found that population-weighted population density (PWPD), some “stay at home” metrics, monthly temperature and precipitation, race/ethnicity, and chronic low-respiratory death rate, were all statistically significant. Of these, PWPD and mobility metrics dominated. This suggests that the biggest impact on COVID-19 deaths was, at least initially, a function of where you lived, and not what you did. However, clearly, increasing social distancing has the net effect of (at least temporarily) reducing the effective PWPD. Our results strongly support the idea that the loosening of “lock-down” orders should be tailored to the local PWPD. In contrast to these variables, while still statistically significant, race/ethnicity, health, and climate effects could only account for a few percent of the variability in deaths. Where associations were anticipated but were not found, we discuss how limitations in the parameters chosen may mask a contribution that might otherwise be present.

## Introduction

Following the first signs of its presence in December 2019 [1], COVID-19 has rapidly spread across the world, impacting almost every country in its wake [2]. Now, more than a year later, while many facets of transmission are better understood, many others remain poorly known (e.g., [3–6]) Its impact has been markedly different in different locations, a consequence of factors that remain actively debated. We can categorize these factors as follows. First, imposed societal interventions, in the form of social distancing measures (e.g., “shelter in place” orders [7], self-isolation [8], school closures [9], etc.). Second, demographic differences in the population including age distribution [10], health [11], gender [12], and race [13] of the populations. Third, climatic variability (e.g., temperature, precipitation, or humidity variations [14]), and spatial heterogeneity in terms of the density of people that a person is typically exposed to [15]. Fourth, environmental variability such as pollution [16]. Fifth, virus connectivity factors, such as the likelihood that the disease will be imported into an area from outside [17]. These are not mutually exclusive categories, and, indeed, we would anticipate a number of confounding variables amongst them.

Our objective in this study is to assess which of these explanatory (independent) variables are clearly important for predicting COVID-19 deaths, primarily to aid in the development of mechanistic model refinements [18–22]. Mechanistic models, as opposed to statistical or machine-learning models are typically developed by incorporating the minimum number of underlying processes necessary to describe the observations sufficiently well to answer specific questions. The types of questions being asked about COVID-19 include, but are not limited to: What impact will interventions have on the trajectory of the disease? Will weather conditions cause the disease to (at least temporarily) abate/increase as we approach the northern summer/winter? Which locations are likely to suffer the most severe outcomes? Which populations will likely fair better, and which will fair worse? A crucial step in answering these questions using models, then, is to understand what factors appear to matter.

## Methodology

### Research design

We developed an analytical, observationally based study by assembling a dataset of parameters that capture, at least in principle, factors likely to modulate the number of COVID-19 deaths. The primary criteria for inclusion were that the data should broadly describe behaviour, age, health, race, climate, and spatial demographics (population density), all of which have been discussed as contributory factors for respiratory diseases. The data, however, were illustrative rather than exhaustive. Potential datasets were excluded if not publicly or readily available.

We focus on the initial rise in cases and deaths from the beginning of March 2020 through mid-May 2020. During this period, populations were essentially entirely susceptible to the disease, and complications arising from the effects or interactions of different factors would be minimised. We perform several multiple-linear-regression (MLR) and machine learning (ML) analyses on the data to identify the key explanatory variables that are likely driving the variations in the number of deaths per 100,000 for the 50 contiguous U.S. states (i.e., their statistical significance). Additionally, we assess the degree to which these variables can explain the observed deaths (i.e., their importance). Our approach for inclusion was liberal: Any dataset that was readily and publicly available and, at least in principle related, was initially included for analysis. However, we designed the study to be illustrative, rather than exhaustive. Thus, where datasets were potentially relevant, but were likely accounted for by another variable, they were excluded. The data tended to fall into two main categories: those that drove (or reduced) transmission and those that impacted the health outcomes of infectious individuals. We identified a range of variables that contributed to these two broad categories.

### Data

Data were collected from a variety of sources, each chosen as a fiduciary of one of the factors described above. They are summarised in Table 1). Briefly, the first six parameters (retail, grocery, parks, transit, workplaces, and residential) were scraped from Google’s Mobility Reports on April 20 [23], and represent the fractional change (expressed as a percentage) in people’s travel to these locations. Thus, a mean value of 11.38% in ‘residential’ represents the increase in people remaining at home since the start of “shelter in place” orders. Similarly, a decrease of 46.58% in ‘retail’ reflects the drop in people going to retail locations. Many of the parameters are self-explanatory, including: age, % 65 years and over [24], obesity rates [25], chronic lower respiratory death rates [26], pollution indices [27], the date that a state of emergency was declared [28], average relative annual and spring-time humidity, dew point, temperature, precipitation [29, 30], and UV index [31]. However, several require further explanation.

**Table 1:** List of parameters used in multiple-regression analysis, together with their basic statistical properties. See text for detailed explanation of each parameter.

Statistic	N	Mean	St. Dev.	Min	Pctl(25)	Pctl(75)	Max
Retail	50	−46.580	6.081	−66	−50	−42.2	−36
Grocery	50	−17.380	7.982	−34	−23.8	−11	1
parks	50	16.400	42.524	−70	−6	29.8	134
Transit	50	−48.520	13.167	−76	−58	−37.5	−21
workplaces	50	−37.760	5.336	−55	−41.8	−35	−27
Residential	50	11.380	2.147	8	10	12	16
Age	50	38.616	2.328	31.000	37.325	39.600	44.900
Low indust. toxins	50	25.500	14.577	1	13.2	37.8	50
Low poll. health risk	50	25.500	14.577	1	13.2	37.8	50
Chron. low resp. death rate	50	42.790	10.520	20	34.9	48.7	64
≥65 years old	50	16.506	1.926	11	15.7	17.5	21
Race param. 1	50	11.954	9.686	0.900	4.850	15.975	38.900
Race param. 2	50	5.580	8.046	1.100	2.325	5.775	56.800
Race param. 3	50	12.070	10.469	1.400	5.125	13.975	49.100
Race param. 4	50	76.164	12.881	24.300	67.825	85.000	94.300
Obesity rates	50	27.740	3.305	19.000	25.600	29.600	35.200
Av. rel. humidity	50	67.102	8.346	38.300	65.950	71.475	77.100
Av. dew point	50	41.710	9.224	26.500	34.925	46.750	65.200
Av. ann. temp. (C)	50	11.074	4.830	−3.000	7.325	14.800	21.500
Av. ann. precip. (mm)	50	941.820	371.752	241	622.5	1,216.2	1,618
State of emerg. dec.	50	−3.100	3.666	−13	−4.8	0	3
Av. spring temp.	50	10.550	4.941	−4.100	6.650	14.050	21.100
Av. spring precip.	50	82.840	34.067	20	56.8	104.5	151
Rel. hum. (morning)	50	76.280	8.593	43	73.2	81	90
rel. hum. (afternoon)	50	48.440	9.004	17	47	53.8	61
UV Index	50	7.780	2.376	1	7	9	12
Deaths/100k (*n*_*deaths*_)	50	20.769	52.440	0.092	1.313	18.250	349.854
PWPD	50	3,298.370	4,113.740	694.900	1,356.450	3,561.875	28,161.500
Date of first death	50	18,341.040	6.465	18,331	18,337	18,346	18,365

Population-weighted population density (PWPD, also known as population-weighted density, PWD) is, the average of each resident’s census tract density, effectively, the density at which the average person lives. It is important to distinguish this from the average population density for a location if there is any significant heterogeneity in population density. For example, the population density of the entire U.S. is approximately 90 people per square mile. In contrast, the population-weighted density is more than 5,000 people per square mile. These data were derived from the WorldPop dataset [32].

The reported date of the first death in each state is used as a proxy for initial seeding of the virus in each state, and, by extension, a proxy for the connectivity of the state to likely sources of the disease (e.g., China, Europe, or other states).

Race parameters 1 through 4 are an attempt to identify any possible links with the underlying races/ethnicities within each state [24]. Specifically, they are: (1) Percent Estimate-Race alone or in combination with one or more other races-Black or African American; (2) Percent Estimate-Race alone or in combination with one or more other races-Asian; (3) Percent Estimate-Hispanic or Latino (of any race); and (4) Percent Estimate-One race-White [33].

**Fig 1:**
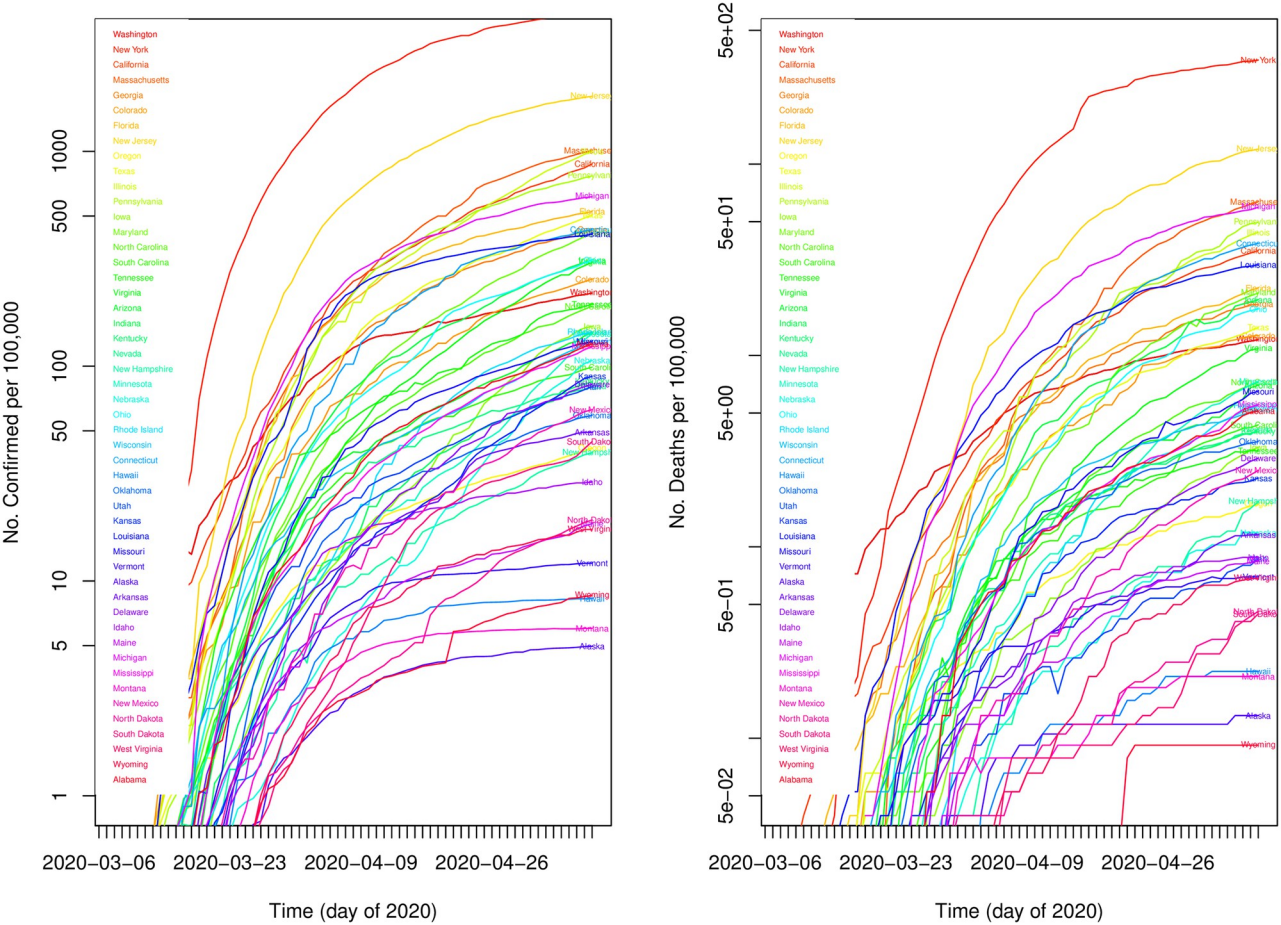
The variation of (Left) Cumulative number of confirmed cases per 100,000 for each state (colored arbitrarily to better separate each curve). (Right) Cumulative number of deaths per 100,000 for each state. Data runs from 2020–03-06 through 2020–05-10.

Finally, our response (dependent) variable is the number of deaths per 100,000 [34]. All values reported here are correct through May 10 2020. This is, of course, an ever-changing value; however, both the cumulative number of confirmed cases as well as the number of deaths per 100,000 for each state tended to maintain similar relative slopes (Fig 1), such that the results do not depend sensitively on the precise date chosen to represent the number of deaths in each state. In fact, the analysis was repeated for five different dates from April 25 through May 10 with no qualitative difference in the results, or their interpretation. It is, nevertheless, worth noting several outliers. Washington, in particular, was the first state to report an appreciable number of deaths in mid-March; however, its initial slope remained shallower than other states, and, by early April, became even flatter. The net effect of this is that in terms of relative number of deaths, from early April to early May, it exchanged position with a dozen other states. The relative positions of other states has also changed during April—May, 2020. In general, however, the changes were likely driven by a particular event that changed the evolving slope of the profile at some point. These typically occurred in the early portion of the outbreak, and the relative positions of the states in May 2020 remained largely unaltered.

### Models

Multiple regression models were analysed using several packages in *R*, primarily relying on ‘lm’ [35]. To identify the explanatory variables that provided the best performing model (i.e., one that lowered the prediction error), we used the ‘stepAIC’ function from the MASS package [36]. Both forward selection and backward selection (i.e., backward elimination) were performed [37]. In the former, initially, no predictors are included in the model, and the algorithm iteratively adds the most contributory variables, stopping when the improvement is no longer statistically significant.

These regression techniques, however, only identify the statistical significance of the variables. To estimate the relative importance of each explanatory variable in describing the variability in deaths, we applied a number of traditional statistical and more modern machine learning (ML) approaches. Using multiple approaches is important for assessing the uncertainty that should be ascribed to a particular ordering of the variables, since different techniques rely on different metrics for importance. The techniques applied included: random forest [38], Xgboost [39], relative importance [40], earth [41], step-wise regression [42], and DALEX [43]. It is important to underscore that these techniques use different definitions of what signifies“important”, and, thus, we do not expect agreement between the results. Nevertheless, where the results do agree is where we can be most confident that the explanatory variable importance is significant, and where they do not, we must remain more cautious.

## Results

When a multiple linear regression analysis was performed on the 28-parameter dataset, four explanatory variables were found to be significant: retail, average annual precipitation, average spring precipitation, and PWPD (S1 Table). Together, *R*^2^ for these variables was 0.973, and thus, these variables are capable of explaining 97% of the variations in deaths/100,000 (*n*_*deaths*_). We then applied a heuristic recursive process of elimination to each of the parameters to identify those that, under a more focused analysis might raise to the point of significance. From this, we identified five explanatory variables: retail, grocery, PWPD, average spring temperature and precipitation. Thus, annual precipitation was no longer deemed to be significant, but grocery and average spring temperature now were (S2 Table).

Comparison of the five most significant variables with one another showed several illuminating features (S1 Fig). First, the number of deaths per 100,000 were clustered below 100 per 100,000, with New York being a significant outlier. Second, unsurprisingly, retail and grocery mobility metrics appear to be the most highly correlated of the explanatory variables. Third, the best fit curve to the number of deaths per 100,000 and PWPD appears to (1) be linear, and (2) display the strongest correlation with *n*_*deaths*_.

An analysis of the residuals suggests that Hawaii, New Mexico, and Rhode Island (observations 11, 31, and 39) deviate most (S2 Fig (left)). The fitted line through the residuals suggest the possibility that a non-linear relationship might be more appropriate; however, given the relatively high *R*^2^ ∼ 0.9, we suggest that this is not necessarily so. Comparing the standardised residuals against theoretical quantiles suggests that the errors are approximately normally distributed, at least for most of the states. Notably, New York (32) and Hawaii (11) (and, to a lesser extent, Rhode Island (39)) are outliers (S2 Fig (right)). To check for homoscedasticity, that is, the assumption of equal variance, we can assess whether the residuals are spread equally along the ranges of the predictors. The notable outliers are, once again, Hawaii (11), Rhode Island (39), and New York (32). Although there is variability, with the exception of New York, the standardised residuals are roughly equal to one and constant along the fitted values (S3 Fig (left)).

Finally, considering the variation of the residuals against leverages, we can estimate to what extent the outliers are influential in the regression analysis. The leverage of a particular observation (state) is related to how much its value on the predictor variable differs from the mean of the predictor variable. We use Cook’s distance as a measure of the influence of an observation, with values lying in the upper-right or lower-right being indicative of unduly influential observations. Unsurprisingly, New York (32) is clearly influential (S3 Fig (right)). Following this, Hawaii (11), and to an even lesser extent, New Jersey are leveraging the results.

**Fig 2:**
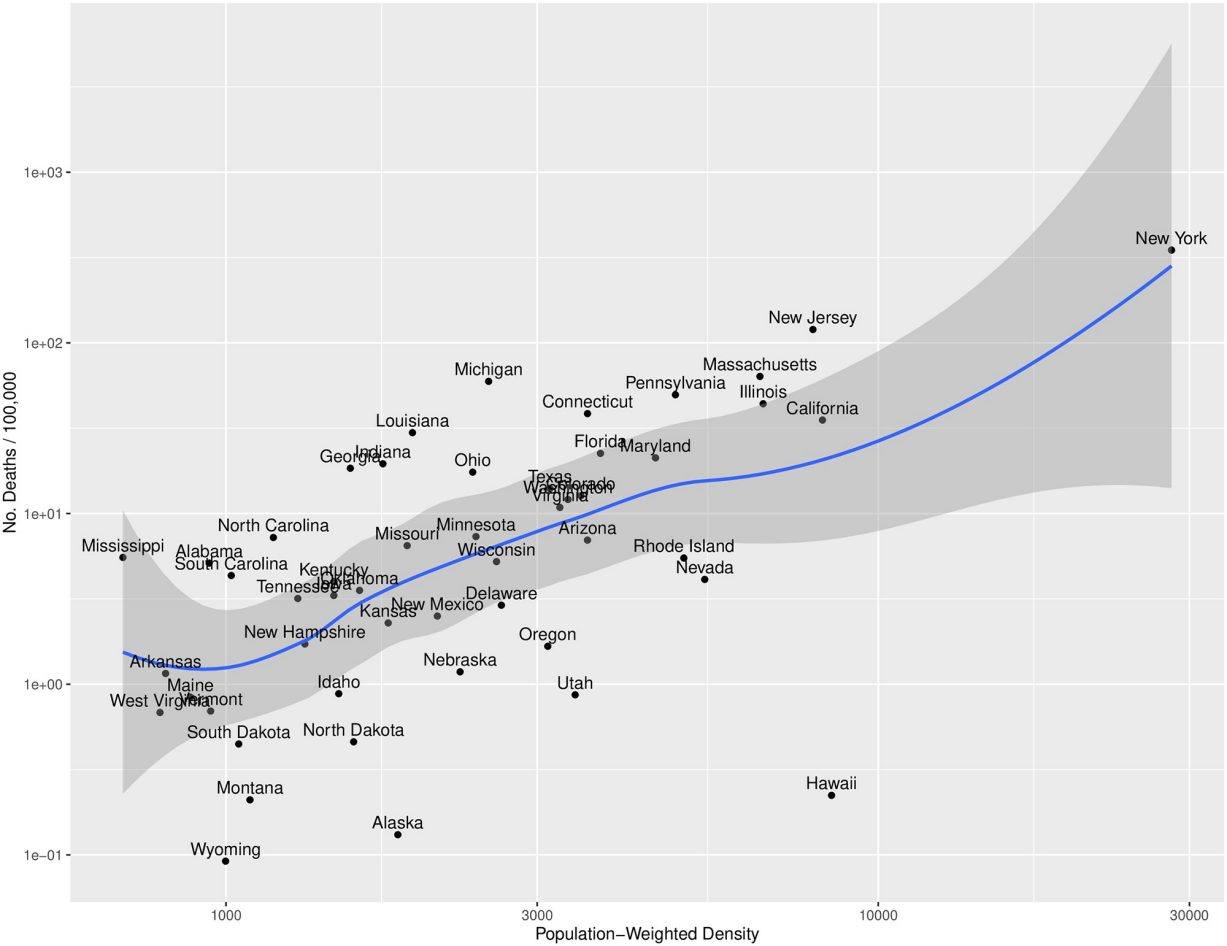
The relationship between the number of deaths per 100,000 and PWPD. Each state is identified with a dot and the variability and smooth profile are shown with the dark grey region and blue line, respectively.

In summary, based on this exploratory analysis, the strongest explanatory variable for deaths per 100,000 is PWPD (Fig 2). There is a clear trend for states with increasing PWPD to have higher number of deaths. There is, however, substantial scatter away from the smooth line through the data, at least in part due to the contribution(s) from some of the other explanatory variables (as well as potentially other equally, or more important variables not included). For example, it is worth noting that, geographically, the states substantially below the line are located at higher latitudes and away from either coast (N. and S. Dakota, Wyoming, Utah, Nebraska, Nevada). On the other hand, many of the states substantially above the line are from southern regions (Mississippi, Alabama, S. Carolina, Kentucky, Missouri, Louisiana). It could also be argued that this demarcation is reflective of other factors, including political affiliation, which may have modulated the timing and/or efficacy of the “shelter in place” orders. Finally, we remark that Hawaii, and to a lesser extent (because of the log-log scale) Alaska, are unique outliers in terms of unusually low deaths given their relative PWPD.

While the preceding analysis heuristically investigated the possible contribution of the explanatory variables in describing the variations in *n*_*deaths*_, we next apply more robust techniques using a variety of algorithms to assess both statistical significance and importance. In these approaches, explanatory variables are iteratively added or removed to identify the subset of variables that produce the best performing model, that is, the model with the lowest prediction error.

First, using *R*’s MASS package, we applied both forward selection, where variables were added iteratively, until the improvement is no longer statistically significant, and backward elimination, where variables are iteratively removed until the point is reached where all the variables are statistically significant (S3 Table). Based on this analysis, 14 variables can account for 97% of the variability in *n*_*deaths*_. This is approximately the same as including all 28 variables, and substantially higher than the 87.5% that we computed using our *ad hoc* approach of searching through the variables. PWPD, race parameter 1, average annual temperature and precipitation, average spring precipitation are the strongest contributors.

Using the Random Forest method to assess the importance of the variables, we found (in order of importance): PWPD, retail, chronic low-respiratory death rate, residential, race parameter 3, obesity rates, average spring temperature, average dew point, race parameter 1, and date of first death were the most important (S4 Table). Comparing this list with the significant variables identified earlier, suggests that any subset of significant *and* important explanatory variables would include at least PWPD, retail, chronic low-respiratory death rate, race parameter 1, and date of first death.

Applying the Xgboost method for ranking explanatory variables in order of their importance identified the ‘retail’ mobility metric as the most important variable, followed by PWPD, and then two more mobility metrics (workplaces and grocery). Race parameter 1, relative humidity (afternoon), date of first death, and average spring temperature and dew point followed. Finally, Chronic low-respiratory death rate was the remaining variable but only 1% as important as the most important variable (S5 Table).

The Multivariate Adaptive Regression Splines (MARS) model can also be used to rank explanatory variables. Unlike the random forest, it has been shown to be more susceptible to unstable explanatory variables. Nevertheless, it is a flexible technique and is included here to provide evidence for the sensitivity of our results to the technique implemented. PWPD, race parameter 2, and retail captured more than 90% of the importance (S6 Table), with grocery and average annual precipitation rounding out the top-five variables.

The step-wise regression method can also be combined with the Akaike Information criteria (AIC) to identify the best model, that is, the best combination of parameters to explain the output variable. Using this approach, in order of importance, PWPD, grocery, retail, average spring temperature and precipitation were all found to be important (S7 Table).

**Fig 3:**
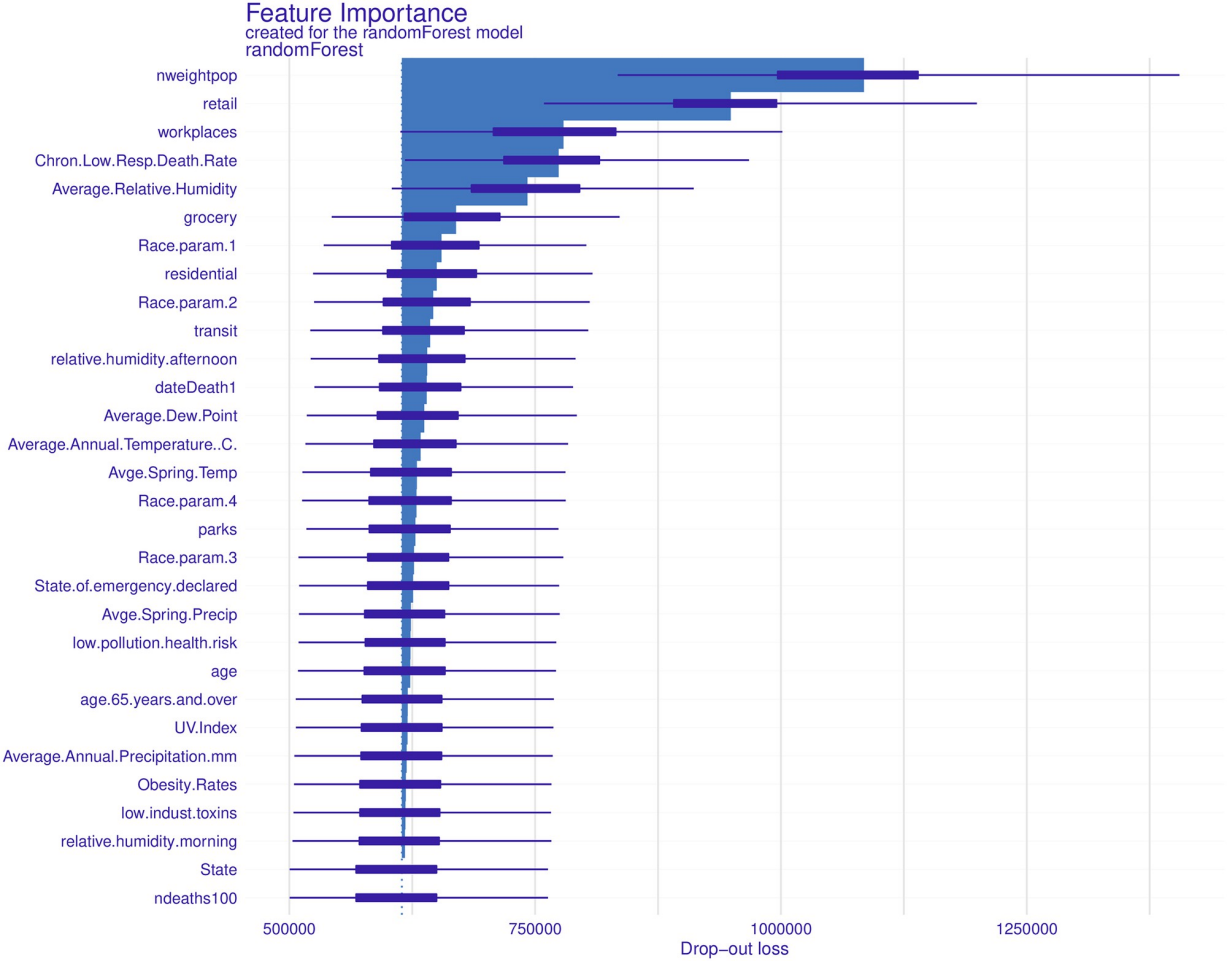
Relative importance of explanatory variables using the DALEX method. See text for more details.

Finally, we consider the DALEX package, which is, in fact, a meta-package in the sense that it can compare responses from different models to allow for direct comparison. Here, though, we apply the DALEX machinery with the random forest technique and use the ‘explain’ and ‘variable_importance’ functions to quantify their relative importance (S8 Table). PWPD, retail/workplaces, chronic low respiratory death rate, average relative humidity, grocery, and race parameter 1 describe most of the observed variability in *n*_*deaths*_. The degree to which these variables contribute to the overall variations can also be visualised graphically (Fig 3). Thus, we infer that the first four variables account for most of the observations. Of these, however, average relative humidity was not found to be statistically significant in any of our analyses. Thus, while humidity was “important”, it was not “significant”.

## Discussion

In this study, we have presented an illustrative, but not exhaustive multiple-regression analysis of 28 explanatory variables, in an attempt to identify those parameters that can best predict deaths per capita in U.S. states. Our results are not surprising in the sense that we would have anticipated that PWPD and lockdowns, in particular, would likely explain at least some of the variations in deaths. It is also reassuring that our results support other studies highlighting the association of race/ethnicity [44], climatic conditions [45], and population demographics [46] with deaths. In fact, given the relative homogeneity of the U.S. population, it is somewhat remarkable that small variations from state to state would result in measurable and statistically-significant results.

Our results suggest that interventions (e.g., social distancing) should be implemented on local scales, not state or even county levels. They should be based on, or at least informed by the PWPD in the area. As the initial disparity between deaths in, say, New York (urban) and Wyoming (rural) underscored, it is reasonable to generate advice for one community that is markedly different than for another community. While our analysis has focused on state-level data, we anticipate that the inferences drawn would apply at even final spatial granularity. Of course, broad policy decisions concerning COVID-19 strategies are multi-factorial and require the consideration of a number of other issues, including economic impacts and the likelihood of adherence by the population. Nevertheless, we believe that PWPD should be an important component of any mitigation strategy.

Our initial dataset contained a number of other variables that were dropped from the formal analysis, primarily because, although there might be an obvious association with deaths, there was no conceivable driving mechanism (i.e., causal relationship). For example, we found that the political affiliation of each state (as measured by the results of the 2016 election) was a strong and statistically significant explanatory variable. In addition to this simply being a confounding variable for several others, such as PWPD (republican states tend to be more rural) and the efficacy of lockdowns (democratic states typically implemented orders earlier and/or more stringently), its variation serves no useful role in explaining the number of deaths. Nevertheless, we emphasise the point here to highlight the dangers in over-interpreting what, based on the analysis presented here, can only be considered associations.

Our study is not without potentially significant limitations. First, our analysis focused on the three-month period at the beginning of the pandemic. As such, given the significant advances since then, in terms of behaviour modification, patient treatment, and vaccines, the relative importance of these explanatory variables may have changed. Thus, strictly, our results should be viewed as being most applicable during the emergence of a novel pathogen.

Second, the datasets analysed were illustrative rather than comprehensive. While other variables may have been more accurate, or contributed more to understanding the variation in deaths, they were not available at the required population level at the time of analysis. For example, underlying Cardiovascular disease (CVD) and diabetes mellitus (DM) are associated with significantly worse outcome in COVID-19 patients [47, 48]. Thus, the lack of inclusion of these co-morbid conditions is a notable limitation. In particular, it is quite possible that some variables attempting to capture, say, the underlying health of the population, may be more important than a variable attempting to capture an environmental condition, such as humidity. And, in particular, for variables that do not contribute substantially, we cannot make general statements about relative importance that, for example, ambient weather conditions play a greater role than, say, health of the population. On the other hand, PWPD undoubtedly plays a major role, and, other variables, like race and climate, do make significant contributions.

Third, the statistical models implemented here are relatively complex. While they have all been rigorously tested and validated, they rely on different assumptions and approximations, and thus, to a modest extent, arrived at slightly different results. However, while this could be viewed as a limitation, we believe that to apply many of them to this problem serves to strengthen the results. Where the same explanatory variables appear in roughly the same position through multiple techniques, we have added confidence that the results are robust. Where not the case, this serves to provide an additional caveat to treat the results with caution.

Making the leap from associations to causal relationships can be accomplished by mathematical models. Thus, we suggest that the results presented here can be further tested within various types of mechanistic models. Indeed, the two major associations of PWPD and mobility metrics are, to varying degrees, already incorporated into models. Individual-based spatial models, for example, can explicitly account for the population-weighted density of the population as well as (at least parametrically) the mobility of people [49]. The significance of climate (particularly temperature, precipitation, and/or humidity) can readily be incorporated as well [20–22].

It is not surprising that PWPD was the most important explanatory variable. It also must be recognised that this variable is related to each state’s social distancing orders, with the primary difference that PWPD is generally immutable, whereas social distancing can and will change over time. It may be tempting, particularly for vulnerable individuals to move to a region of lower PWPD, as this will likely have a strong impact on their likelihood of dying from COVID-19. It could be argued that this should be balanced with the likelihood of the quality of healthcare being proportionately worse in more rural areas. However, based on the simple, but clear relationship between the number of deaths per 100,000 and PWPD, this appears not to be the case: Regions of lower PWPD experienced lower deaths per capita.

Although it is intuitively obvious that PWPD and *n*_*deaths*_ should be positively correlated in terms of causality, increases in PWPD would primarily drive higher incidence, which would, in turn, result in more deaths. However, an interesting question not addressed here is whether the relationship between PWPD and deaths is linear? If a disproportionate number of more vulnerable people live in higher-PWPD regions, this would result in a plot of *n*_*deaths*_ versus PWPD turning upwards. There is some suggestion of this in our results (Fig 2); however, such an inference is at best tentative and requires more detailed analysis, including additional datasets that are likely not yet available.

The explanatory variables analysed in this study can be categorised in a number of ways. One important distinction that can be made are those variables that impact the transmission of the virus from one individual to another (e.g., movement variables, such as ‘retail’ and ‘grocery’, or environmental variables such as humidity, precipitation, or temperature) and those that attempt to capture the relative vulnerability of the individual (e.g., age, race, and obesity demographics). While the former directly impact the number of cases of COVID-19, which then drive the number of deaths, the latter affect only the patient’s outcome once they have contracted the virus.

It was somewhat surprising that no significance was found for any measures of age, chronic disease, pollution, or some of the race parameters. In some cases, this is likely due to the fact that the variability was just too small to drive a signal in the results. For example, while the average obesity rate across the 50 states is 27.7%, the standard deviation is only 3.3%. In these cases, we suggest that rather than there not being contribution, we were unable to identify it because of the quality of the parameter. Indeed, several recent studies, which have focused specifically on a particular parameter, such as race or ethnicity, were able to identify it within smaller populations with higher heterogeneity [50]. Additionally, where explanatory variables are co-linear, the explanatory capability might be absorbed by another parameter.

Equally remarkable is that small differences in race/ethnicity amongst states were statistically significant, albeit only accounting for a small fraction in the variability of the deaths. This suggests that these factors may be more important than those that were not found to be statistically significant here. Indeed a number of studies have uncovered the strong association between race/ethnicity and COVID-related deaths (e.g., [51]).

To better understand the role of these explanatory variables, we focused on the initial three month period during the spring of 2020. Now, almost a year later, the situation has become substantially more complex, with variables interacting with one another in subtle ways. In particular, when deaths rose substantially in certain regions, this drove the population to adapt its behaviour through the application of better social distancing and/or mask use. This occurred both locally where the increase in mortality occurred as well as remotely based on people’s recognition of it. Given the sequence of waves that have already occurred over the last twelve months, the distribution of vaccines in the upcoming months, and the emergence of new variants, it is likely that understanding what are the primary factors driving COVID-19 deaths will remain a key question for the foreseeable future.

## Supporting information

S1 Fig Scatterplot matrix of five significant explanatory variables for the number of deaths per 100,000 in each state. Panels show: Number of deaths per 100,000, retail mobility index, grocery mobility index, PWPD, average spring temperature, and average spring precipitation. Data are shown by the circles, regression lines are solid, smoothed mean values are shown by the dashed line, and variances are shown b the dashed-dotted lines.
(PDF)

S2 Fig (Left) Residuals versus fitted values. (Right) Q-Q plot of standardised residuals. Used as a diagnostic, the residuals versus fitted plot should be approximately random, while the Q-Q plot (a normal probability plot) should be approximately straight if the errors are distributed normally.
(PDF)

S3 Fig (Left) Scale-location. (Right) Residuals versus leverage. As a diagnostic, the points in the scale-location plot should be distributed randomly, while the last residuals versus leverage suggests which point have the greatest influence on the model. See text for more details on interpretation.
(PDF)

S1 Table Summary of multiple-regression analysis on 28 explanatory variables described in Table 1. (PDF)

S2 Table Summary of the five most significant explanatory variables for predicting the number of deaths per 100,000 in each state. (PDF)

S3 Table Step-wise regression using the MASS package. (PDF)

S4 Table Random forest method for relative importance of parameters. (PDF)

S5 Table Xgboost method for relative importance of parameters. Top-20 shown.
(PDF)

S6 Table MARS method for relative importance of parameters. (PDF)

S7 Table Step-wise regression method for relative importance of parameters. (PDF)

S8 Table Step-wise regression method for relative importance of parameters. (PDF)
