# Supplementary material for: COVID-19 deaths: Which explanatory variables matter the most?
Source: PLoS One. 2022 Apr 21;17(4):e0266330. doi: 10.1371/journal.pone.0266330 (PMC9022803; doi:10.1371/journal.pone.0266330)
Supplement: S1 Fig — Panels show: Number of deaths per 100,000, retail mobility index, grocery mobility index, PWPD, average spring temperature, and average spring precipitation. Data are shown by the circles, regression lines are solid, smoothed mean values are shown by the dashed line, and variances are shown b the dashed-dotted lines. (PDF) [file pone.0266330.s001.pdf]

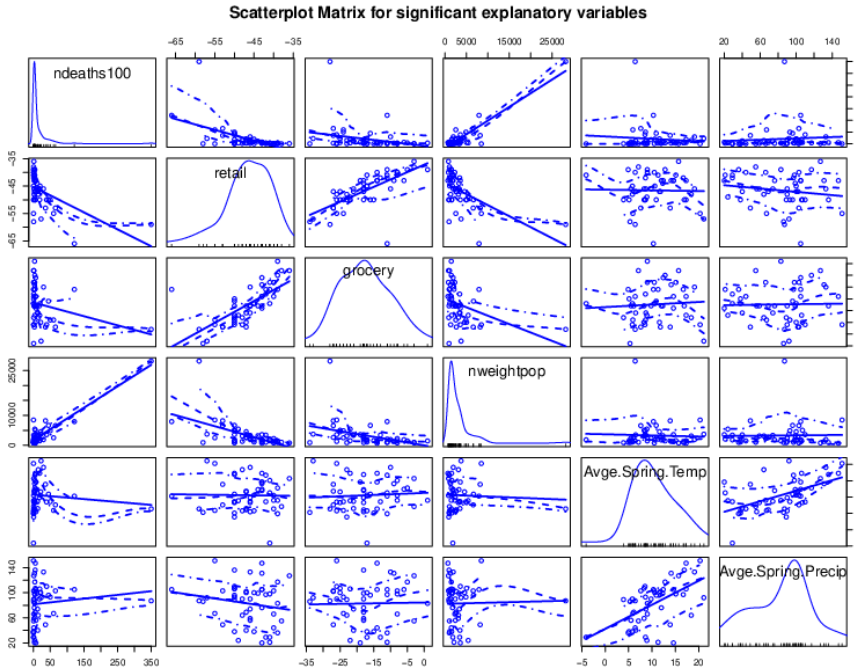

Figure S1: **Scatterplot matrix of five significant explanatory variables for the number of deaths per 100,000 in each state.** Panels show: Number of deaths per 100,000, retail mobility index, grocery mobility index, PWPD, average spring temperature, and average spring precipitation. Data are shown by the circles, regression lines are solid, smoothed mean values are shown by the dashed line, and variances are shown b the dashed-dotted lines.
