# Supplementary material for: COVID-19 deaths: Which explanatory variables matter the most?
Source: PLoS One. 2022 Apr 21;17(4):e0266330. doi: 10.1371/journal.pone.0266330 (PMC9022803; doi:10.1371/journal.pone.0266330)
Supplement: S2 Fig — (Left) Residuals versus fitted values. (Right) Q-Q plot of standardised residuals. Used as a diagnostic, the residuals versus fitted plot should be approximately random, while the Q-Q plot (a normal probability plot) should be approximately straight if the errors are distributed normally. (PDF) [file pone.0266330.s002.pdf]

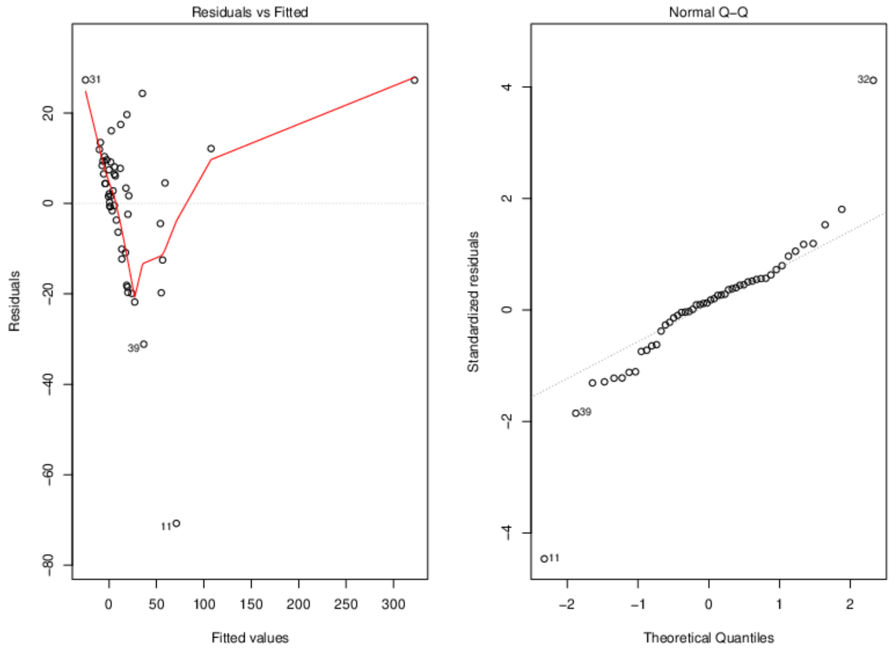

Figure S2: **(Left) Residuals versus fitted values. (Right) Q-Q plot of standardized residuals.** Used as a diagnostic, the residuals versus fitted plot should be approximately random, while the Q-Q plot (a normal probability plot) should be approximately straight if the errors are distributed normally.
