# Supplementary material for: COVID-19 deaths: Which explanatory variables matter the most?
Source: PLoS One. 2022 Apr 21;17(4):e0266330. doi: 10.1371/journal.pone.0266330 (PMC9022803; doi:10.1371/journal.pone.0266330)
Supplement: S3 Fig — (Left) Scale-location. (Right) Residuals versus leverage. As a diagnostic, the points in the scale-location plot should be distributed randomly, while the last residuals versus leverage suggests which point have the greatest influence on the model. See text for more details on interpretation. (PDF) [file pone.0266330.s003.pdf]

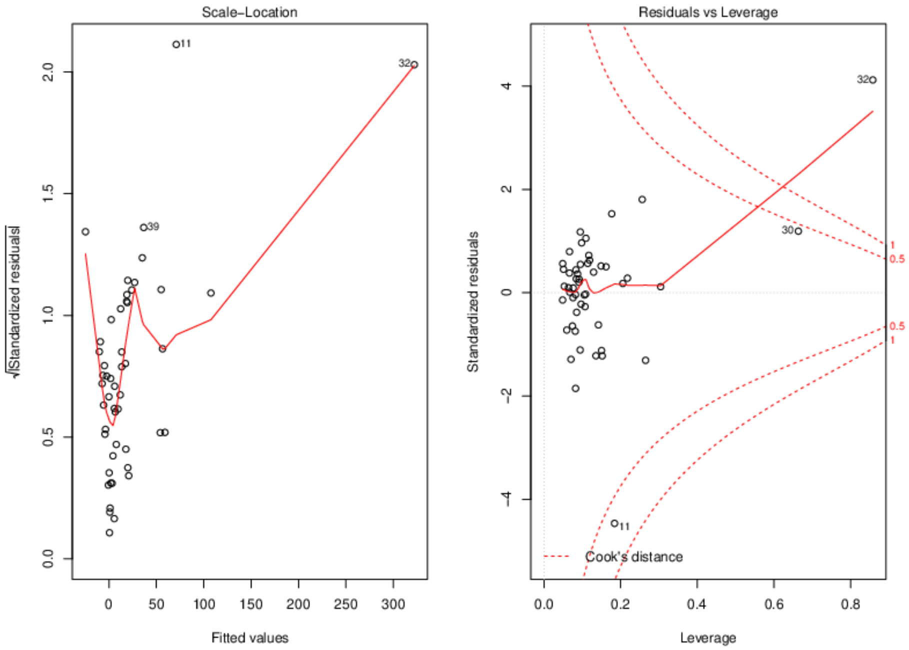

Figure S3: **(Left) Scale-location. (Right) Residuals versus leverage.** As a diagnostic, the points in the scale-location plot should be distributed randomly, while the last residuals versus leverage suggests which point have the greatest influence on the model. See text for more details on interpretation.
