# Supplementary material for: COVID-19 deaths: Which explanatory variables matter the most?
Source: PLoS One. 2022 Apr 21;17(4):e0266330. doi: 10.1371/journal.pone.0266330 (PMC9022803; doi:10.1371/journal.pone.0266330)
Supplement: S1 Table — (PDF) [file pone.0266330.s004.pdf]

Table S1: Summary of multiple-regression analysis on 28 explanatory variables described in Table 1.

	Dependent variable:
	ndeaths100
retail	−2.545*** (0.870)
grocery	0.842 (0.579)
parks	0.085 (0.097)
transit	0.665 (0.587)
workplaces	−0.505 (0.930)
residential	−1.852 (4.689)
age	−2.736 (4.279)
low.indust.toxins	−0.146 (0.310)
low.pollution.health.risk	0.056 (0.262)
Chron.Low.Resp.Death.Rate	0.639 (0.545)
age.65.years.and.over	2.218 (4.373)
Race.param.1	1.405 (1.181)
Race.param.2	0.286 (1.538)
Race.param.3	0.292 (0.553)
Race.param.4	0.641 (1.011)
Obesity.Rates	0.137 (1.373)
Average.Relative.Humidity	−0.413 (0.435)
Average.Dew.Point	−0.118 (0.418)
Average.Annual.Temperature..C.	−4.991 (7.511)
Average.Annual.Precipitation.mm	0.106** (0.043)
State.of.emergency.declared	0.906 (0.862)
Avge.Spring.Temp	2.155 (7.214)
Avge.Spring.Precip	−0.911** (0.411)
relative.humidity.morning	−0.320 (0.620)
relative.humidity.afternoon	0.308 (0.617)
UV.Index	2.239 (1.576)
PWPD	0.012*** (0.001)
dateDeath1	−0.692 (0.636)
Constant	12,608.070 (11,656.840)
Observations	50
R^2	0.973
Adjusted R^2	0.937
Residual Std. Error	13.136 (df = 21)
F Statistic	27.140*** (df = 28; 21)
Note:	*p<0.1; **p<0.05; ***p<0.01
