# Supplementary material for: COVID-19 deaths: Which explanatory variables matter the most?
Source: PLoS One. 2022 Apr 21;17(4):e0266330. doi: 10.1371/journal.pone.0266330 (PMC9022803; doi:10.1371/journal.pone.0266330)
Supplement: S2 Table — (PDF) [file pone.0266330.s005.pdf]

Table S2: Summary of the five most significant explanatory variables for predicting the number of deaths per 100,000 in each state.

	Dependent variable:
	ndeaths100
retail	-1.625^{**}
	(0.750)
grocery	1.382^{***}
	(0.507)
PWPD	0.011^{***}
	(0.001)
Avge.Spring.Temp	-1.438^{**}
	(0.683)
Avge.Spring.Precip	0.172^{*}
	(0.102)
Constant	-67.055^{**}
	(27.940)
Observations	50
R^2	0.875
Adjusted R^2	0.861
Residual Std. Error	19.544 (df = 44)
F Statistic	61.753^{***} (df = 5; 44)
Note:	$^{*}p<0.1$; $^{**}p<0.05$; $^{***}p<0.01$
