# Supplementary material for: COVID-19 deaths: Which explanatory variables matter the most?
Source: PLoS One. 2022 Apr 21;17(4):e0266330. doi: 10.1371/journal.pone.0266330 (PMC9022803; doi:10.1371/journal.pone.0266330)
Supplement: S3 Table — (PDF) [file pone.0266330.s006.pdf]

Table S3: step-wise regression using the MASS package.

	Dependent variable:
	ndeaths100
retail	-2.389^{***} (0.502)
grocery	0.814^{**} (0.348)
parks	0.107 (0.065)
transit	0.765^{**} (0.306)
Chron.Low.Resp.Death.Rate	0.570^{**} (0.272)
Race.param.1	0.986^{***} (0.284)
Race.param.4	0.460^{*} (0.228)
Average.Relative.Humidity	-0.505^{**} (0.222)
Average.Annual.Temperature..C.	-2.110^{***} (0.670)
Average.Annual.Precipitation.mm	0.096^{***} (0.022)
State.of.emergency.declared	0.828 (0.504)
Avge.Spring.Precip	-0.901^{***} (0.232)
UV.Index	2.208^{**} (0.977)
PWPD	0.012^{***} (0.001)
dateDeath1	-0.692^{**} (0.318)
Constant	$12{,}564.970^{**}$ (5,852.469)
Observations	50
R^2	0.971
Adjusted R^2	0.958
Residual Std. Error	10.723 (df = 34)
F Statistic	75.863^{***} (df = 15; 34)
Note:	$^{*}p<0.1$; $^{**}p<0.05$; $^{***}p<0.01$
