# Supplementary material for: COVID-19 deaths: Which explanatory variables matter the most?
Source: PLoS One. 2022 Apr 21;17(4):e0266330. doi: 10.1371/journal.pone.0266330 (PMC9022803; doi:10.1371/journal.pone.0266330)
Supplement: S4 Table — (PDF) [file pone.0266330.s007.pdf]

Table S4: Random Forest Method for relative importance of parameters.

| Parameter | Overall |
|---|---|
| retail | 6.70932241 |
| grocery | 1.65334776 |
| parks | 0.40933968 |
| transit | -1.05140388 |
| workplaces | -1.63133731 |
| residential | 3.44869838 |
| age | 0.44395140 |
| low.indust.toxins | 0.94348756 |
| low.pollution.health.risk | -1.89831370 |
| Chron.Low.Resp.Death.Rate | 5.42687854 |
| age.65.years.and.over | -1.76708823 |
| Race.param.1 | 1.61188454 |
| Race.param.2 | 1.29263755 |
| Race.param.3 | 2.82938257 |
| Race.param.4 | 0.98131658 |
| Obesity.Rates | 2.08230701 |
| Average.Relative.Humidity | -0.59747647 |
| Average.Dew.Point | 1.72889125 |
| Average.Annual.Temperature..C. | 0.06729476 |
| Average.Annual.Precipitation.mm | 1.02933029 |
| State.of.emergency.declared | -0.79372186 |
| Avge.Spring.Temp | 2.06492880 |
| Avge.Spring.Precip | -1.01229332 |
| relative.humidity.morning | -0.80503322 |
| relative.humidity.afternoon | 3.06231970 |
| UV.Index | 0.68373784 |
| PWPD | 7.16775890 |
| dateDeath1 | 1.48484773 |
