# Supplementary material for: COVID-19 deaths: Which explanatory variables matter the most?
Source: PLoS One. 2022 Apr 21;17(4):e0266330. doi: 10.1371/journal.pone.0266330 (PMC9022803; doi:10.1371/journal.pone.0266330)
Supplement: S5 Table — Top-20 shown. (PDF) [file pone.0266330.s008.pdf]

Table S5: Xgboost Method for relative importance of parameters. Top-20 shown.

Parameter	Overall
retail	100.00000
PWPD	31.50172
workplaces	11.96575
grocery	9.92375
Race.param.1	3.09216
relative.humidity.afternoon	2.92808
dateDeath1	2.57557
Avge.Spring.Temp	1.85285
Average.Dew.Point	1.39623
Chron.Low.Resp.Death.Rate	1.18016
parks	0.72053
low.pollution.health.risk	0.47567
UV.Index	0.38364
Average.Annual.Precipitation.mm	0.23422
transit	0.22316
age.65.years.and.over	0.14004
relative.humidity.morning	0.13864
Race.param.4	0.13657
Race.param.2	0.10647
low.indust.toxins	0.08646
