# Supplementary material for: COVID-19 deaths: Which explanatory variables matter the most?
Source: PLoS One. 2022 Apr 21;17(4):e0266330. doi: 10.1371/journal.pone.0266330 (PMC9022803; doi:10.1371/journal.pone.0266330)
Supplement: S6 Table — (PDF) [file pone.0266330.s009.pdf]

Table S6: MARS Method for relative importance of parameters.

Parameter	nsubsets	gcv	rss
PWPD	6	100.0	100.0
Race.param.2	5	29.0	31.3
retail	4	20.4	22.8
grocery	3	10.9	14.8
Average.Annual.Precipitation.mm	1	4.5	7.4
