# Supplementary material for: COVID-19 deaths: Which explanatory variables matter the most?
Source: PLoS One. 2022 Apr 21;17(4):e0266330. doi: 10.1371/journal.pone.0266330 (PMC9022803; doi:10.1371/journal.pone.0266330)
Supplement: S7 Table — (PDF) [file pone.0266330.s010.pdf]

Table S7: Step-wise Regression Method for relative importance of parameters.

Parameter	Df	Sum of Sq	RSS	AIC
none		16807	302.88	
Avge.Spring.Precip	1	1088	17895	304.01
Avge.Spring.Temp	1	1694	18501	305.68
retail	1	1795	18603	305.95
grocery	1	2842	19649	308.69
PWPD	1	72961	89768	384.65
