# Supplementary material for: COVID-19 deaths: Which explanatory variables matter the most?
Source: PLoS One. 2022 Apr 21;17(4):e0266330. doi: 10.1371/journal.pone.0266330 (PMC9022803; doi:10.1371/journal.pone.0266330)
Supplement: S8 Table — (PDF) [file pone.0266330.s011.pdf]

Table S8: Step-wise Regression Method for relative importance of parameters.

No.	variable	mean dropout loss	label
1	full model	614479.0	randomForest
2	State	614479.0	randomForest
3	ndeaths100	614479.0	randomForest
4	relative.humidity.morning	617600.1	randomForest
5	low.indust.toxins	618002.4	randomForest
6	Obesity.Rates	618421.8	randomForest
7	Average.Annual.Precipitation.mm	619321.7	randomForest
8	UV.Index	620157.1	randomForest
9	age.65.years.and.over	620374.4	randomForest
10	age	622894.6	randomForest
11	low.pollution.health.risk	623242.5	randomForest
12	Avge.Spring.Precip	623606.0	randomForest
13	State.of.emergency.declared	625839.0	randomForest
14	Race.param.3	626778.1	randomForest
15	parks	628155.8	randomForest
16	Race.param.4	629312.3	randomForest
17	Avge.Spring.Temp	629676.5	randomForest
18	Average.Annual.Temperature..C.	633470.7	randomForest
19	Average.Dew.Point	637143.8	randomForest
20	dateDeath1	639675.7	randomForest
21	relative.humidity.afternoon	640153.5	randomForest
22	transit	643169.1	randomForest
23	Race.param.2	646339.4	randomForest
24	residential	649959.4	randomForest
25	Race.param.1	654734.9	randomForest
26	grocery	669505.6	randomForest
27	Average.Relative.Humidity	742232.9	randomForest
28	Chron.Low.Resp.Death.Rate	773875.2	randomForest
29	workplaces	778847.0	randomForest
30	retail	948946.8	randomForest
31	PWPD	1084427.7	randomForest
32	baseline	3952434.6	randomForest
